# Life finds a way *(dedicated to palliative care professionals in distress)*

**DOI:** 10.1017/S1478951525101326

**Published:** 2025-12-29

**Authors:** Bianca Sakamoto Ribeiro Paiva

**Affiliations:** 1 Research Group on Palliative Care and Health-Related Quality of Life, Barretos Cancer Hospital, Barretos, Brazil; 2 Institute of Education and Research, Barretos Cancer Hospital, Barretos, Brazil

When I’m on the other side
the side of me as a person
I often find myself in an emptiness.
Maybe I got lost for a moment,
though I can’t pinpoint exactly when.
But it was in that very same moment
that I found myself again…
distant.
Distant from who I once was,
perhaps closer to who I truly am.
Caring for others feels so natural
I learned this gesture:
the act of compassionate care,
of respecting who the other is,
was, and will be.
But caring for myself…
Ah, that gesture seems to come with many limits.
And it is precisely within those limits
that I realize something has grown too narrow.
Something has become too painful.
Something has broken the boundaries of my own self.
And as I trace back the path
of looking at Life
and at my Self,
I come to see:
Life resists,
Life insists,
Life persists.
It shows me a new path.
Perhaps this new path
won’t allow me to walk as I once did
but in a way that is more
meaningful,
resilient.
A path where hope
is sometimes present
but I don’t feel it,
don’t understand it,
don’t always want to see it.
Only later do I realize:
there is a new way of walking.
Life finds a way.
Life reveals ways.
Life makes me walk
through pain, through suffering, through feeling, and through silence.
Life helps me understand:
I, too, need care.
My Self is just as necessary to me
as the patient is to their physician.
Life shows me the way
to live with more self-compassion.
To live knowing
that I am more important today
than I ever imagined.
Life finds a way.

